# Effect of Crumb Rubber, Fly Ash, and Nanosilica on the Properties of Self-Compacting Concrete Using Response Surface Methodology

**DOI:** 10.3390/ma15041501

**Published:** 2022-02-17

**Authors:** Nurul Izzati Rahim, Bashar S. Mohammed, Isyaka Abdulkadir, Mohammed Dahim

**Affiliations:** 1 Civil and Environmental Engineering Department, Faculty of Engineering, Universiti Teknologi PETRONAS (UTP), Bandar Seri Iskandar 32610, Perak, Malaysia; nurul_18001893@utp.edu.my (N.I.R.); isyaka_18000638@utp.edu.my (I.A.); 2 Civil Engineering Department, Bayero University, Kano 700241, Nigeria; 3 Civil Engineering Department, King Khalid University, Abha 61421, Saudi Arabia; madahim@kku.edu.sa

**Keywords:** self-compacting concrete (SCC), crumb rubber (CR), nanosilica (NS), response surface methodology (RSM)

## Abstract

Producing high-strength self-compacting concrete (SCC) requires a low water-cement ratio (W/C). Hence, using a superplasticizer is necessary to attain the desired self-compacting properties at a fresh state. The use of low W/C results in very brittle concrete with a low deformation capacity. This research aims to investigate the influence of crumb rubber (CR), fly ash (FA), and nanosilica (NS) on SCC’s workability and mechanical properties. Using response surface methodology (RSM), 20 mixes were developed containing different levels and proportions of FA (10–40% replacement of cement), CR (5–15% replacement of fine aggregate), and NS (0–4% addition) as the input variables. The workability was assessed through the slump flow, T_500_, L-box, and V-funnel tests following the guidelines of EFNARC 2005. The compressive, flexural, and tensile strengths were determined at 28 days and considered as the responses for the response surface methodology (RSM) analyses. The results revealed that the workability properties were increased with an increase in FA but decreased with CR replacement and the addition of NS. The pore-refining effect and pozzolanic reactivity of the FA and NS increased the strengths of the composite. Conversely, the strength is negatively affected by an increase in CR, however ductility and deformation capacity were significantly enhanced. Response surface models of the mechanical strengths were developed and validated using ANOVA and have high R^2^ values of 86–99%. The optimization result produced 36.38%, 4.08%, and 1.0% for the optimum FA, CR, and NS replacement levels at a desirability value of 60%.

## 1. Introduction

Self-consolidating concrete (SCC) is defined as concrete that flows freely under its weight and does not require compaction or vibration during casting [1,2]. SCC was pioneered in Japan in the late 1980s and was widely used in civil works by European nations beginning in France in the mid-1990s [3,4]. Many researchers have shown that using SCC improves workability, lowers cement content, improves permeability, and increases the durability of concrete. It is favorable because of its high flow rate, cohesiveness, and good passing ability, allowing it to travel through congested reinforcements [3,5,6]. Furthermore, the SCC demonstrates high flexural and tensile strength than conventional concrete due to its dense microstructure [5] and improved interfacial transition zone between the individual particles [3].

On the other hand, SCC is brittle and has a poor yield strength because of its low W/C and compact microstructure [5,7]. Previous research has demonstrated that the brittle nature of SCC may be mitigated by adding CR into the mix to produce rubberized SCC (R-SCC) [5]. Research on R-SCC has reported that the composite benefits from the combined superior properties of SCC and the improved ductility, deformation capacity, and energy absorption of crumb rubber concrete [3,8]. The use of crumb rubber (CR) in SCC further adds to the existing efforts in recycling waste materials by utilizing them in concrete production for sustainability considerations. Several kinds of research have already been executed on recycling waste materials in SCC, as evident from the recent review works by Gupta et al. [9], Revilla-Cuesta et al. [10], Faraj et al. [11], and Ferrara et al. [12]. CR seems to be very popular among the waste materials utilized due to its multiple advantages to concrete and the environment.

With the increase in population growth, the transportation industries increased the production of vehicles to meet increasing demands, which ultimately led to increased tire production [13,14,15]. The usage of vehicles generates massive stockpiles of waste tires every year. About 1 billion waste tires are generated each year globally, and it is estimated that about 5 billion will be generated by 2030 [14,15]. Tires are made to last longer by incorporating some chemicals into the ingredients, causing them to be non-biodegradable. Furthermore, tires produce toxic leachate to the ground, thus causing pollution to the land and water bodies nearby. Some tires are burnt due to limited storage space, causing air pollution by releasing carbon dioxide into the atmosphere [16,17]. Waste tires are harmful to the environment and human health; hence construction industries find a way to reuse and recycle them to support sustainability in construction [18,19,20].

Fibers and steel from tires are removed before ground to smaller sizes called CR. CR is proposed for use as a partial substitute to fine aggregate in concrete [21,22], asphalt concrete [23], or pavement construction [21] to improve the qualities of the concrete by making it less dense, more robust, ductile, and impact resistant [24]. Furthermore, the use of CR helps reduce the brittleness of the concrete, resulting in a gradual ductile rather than a rapid brittle failure and reducing the excessive depletion of naturally occurring fine aggregate due to increased construction activities caused by rapid urbanization and population growth [13].

However, concrete’s mechanical and durability behaviors are significantly affected by incorporating CR. The strength of the concrete decreases with an increase in CR proportion in the mix [25,26]. Similarly, the size of the CR affects the strength of the concrete, with smaller-sized CR particles leading to lesser mechanical strength loss than bigger-sized particles [27]. Many researchers have opined that the loss in mechanical strengths of concrete by CR inclusion is due to the poor bonding between the CR and hardened cement paste. Furthermore, due to the CR particles’ hydrophobic nature, air becomes trapped on their surface during mixing, which becomes void at a hardened state, increasing the porosity of the concrete. Furthermore, CR decreases the fresh concrete’s workability, flowability, passing ability, and segregation resistance and increases its viscosity [28].

In order to overcome the poor bonding problem, different CR surface pretreatment methods have been proposed, such as the use of NaOH [29,30,31], precoating with silica fume [32,33], and with cementitious materials [30], use of silane coupling agent [34,35,36], and thermal pretreatment [37], among others. Regarding the porosity issue, the use of micro and nanomaterials such as nanosilica, silica fume, and fly ash have been proposed, as reported by several researchers [2,38,39,40,41]. The nanoparticles of the proposed materials tend to refine the pore structure and densify the composite through the filling action and their reactivity as pozzolans. These materials also positively enhance the workability of the fresh mix due to their spherical shape and smooth surface morphology.

The use of nanosilica (NS) to enhance the properties of rubberized cementitious composites in fresh and hardened states is now gaining ground. NS has the same favorable reaction as micro silica or silica fume, and it can improve the strength and durability of concrete, regardless of its size [42]. Shahrul et al. [43] investigated the influence of nanosilica on the properties of rubberized mortar. Their findings revealed a significant improvement in the mechanical properties of rubberized mortar at an optimum NS content of 2.5% by weight of cement. However, it is detrimental at a higher dosage of 5.0%. Similarly, in a preliminary study, some of the current authors had investigated the influence of nanosilica and fly ash on the workability properties of R-SCC. The results indicated that the workability properties of the mix assessed through slump flow, L-box, and V-funnel tests were significantly improved at an optimum NS content of 2% [44]. The reader can refer to the following literature for recent research on the effect of NS and CR on the properties of SCC [44], a combination of NS and silica fume (micro silica) on the mechanical and durability of SCC [45], the effect of CR and silica fume on the mechanical properties and fracture toughness of SCC [46], only nanosilica in SCC [47], predictive models for the mechanical properties of SCC containing CR and silica fume [48], the effect of NS and micro-silica on the drying shrinkage and compressive strength of SCC [49], the effect of silica fume on the durability of SCC [50], the effect of only CR on the properties of SCC [51], and finally, an investigation on the effect of glass fiber, as well as a NS and fly ash (FA) blend on the fresh properties of SCC [52].

Hence, despite the available literature on the use of materials such as FA, NS, SF, and CR in SCC, it is evident that there is no research yet on the combined influence of FA, NS, and CR together on the properties of SCC. Some of the current authors have partially covered some aspects of this topic [44], leaving room for more investigations that this research aims to address. This research aims to investigate, model, and optimize the effects of NS, FA, and CR on the workability and mechanical properties of self-compacting concrete using the response surface methodology (RSM) approach. Development of response-based predictive models of the properties of the SCC and the multi-objective optimization is part of the novelty of this research. The research will effectively employ the three key ingredients harmoniously to make a self-compacting concrete that fulfills the European Federation of National Associations Representing for Concrete (EFNARC) 2005 criteria in a fresh state and has good mechanical characteristics when hardened.

## 2. Materials and Methods

### 2.1. Materials Compositions and Properties

**Figure 1:**
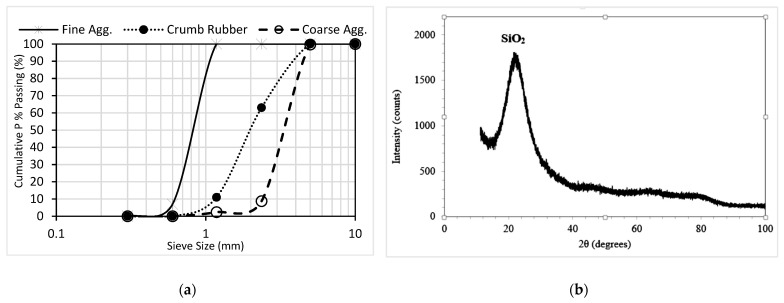
(**a**) Grading of aggregates and CR; (**b**) XRD pattern of nanosilica.

**Figure 2:**
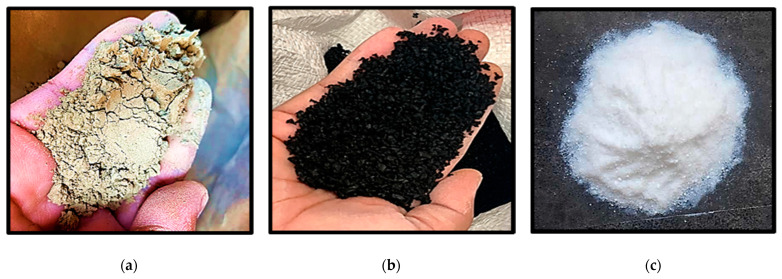
The materials used as the RSM input factors. (**a**) class fly ash; (**b**) crumb rubber, and (**c**) nanosilica.

The materials used in this research include chipping stones and river sand as coarse and fine aggregates, respectively conforming to ASTM C33-18 requirements [53]. The CR used to replace fine aggregate has a particle size of 1–5 mm. The coarse aggregate has an average size of 10 mm, while the fine aggregate range is 0.3 mm to 2.36 mm. Other properties of the aggregates and the grading curves are shown in Table 1 and Figure 1a, respectively. Tasek brand Type 1 ordinary Portland cement(OPC) of grade 42.5 N (4698-W) supplied by Tasek Corporation SDN Berhad, Ipoh, Perak, Malaysia, having specific gravity, loss on ignition, and fineness of 3.15, 0.64, and 440 m^2^/kg, respectively, was used. Class F fly ash (FA) having a specific gravity of 2.38 and loss on ignition of 1.87% was used. The chemical composition of the OPC and FA is presented in Table 2. The NS used is of high quality, having a 99.82% SiO_2_ content, loss on ignition of 6%, specific surface area of 100 ± 25 m^2^/g, PH of 6.5–7.5, surface density of 0.2 g/mL, and an XRD pattern as shown in Figure 1b. The pictures of the FA, CR, and NS used are presented in Figure 2. A Sica Viscocrete-2044 water-reducing superplasticizer was used to achieve the self-compacting properties at a water-binder ratio (W/B) of 0.35. The plasticizer dosage was kept constant at 1.0% for all the mixes.

### 2.2. RSM Mix Design and Materials Proportioning

The RSM input variables considered were the FA (10%, 25%, 40%) replacement of cement by weight, CR (0%, 7.5%, 15%) replacement of fine aggregate by volume, and NS (0%, 2%, 4%) addition by weight of cement. Using the central composite design of the RSM, 20 experimental runs were generated with different combinations of the three levels of the input factors (FA, CR, and NS). The RSM-generated mixes and quantities of materials used are presented in Table 3. A constant coarse aggregate and W/B of 588 kg/m^3^ and 0.35 were used in all the mixes. Experimental investigations on the mixes were carried out to determine the responses under consideration. The workability properties of the concrete were assessed through the slump flow (flow diameter and T_500_), L-box, and V-funnel tests. At the hardened state, the responses considered were the compressive, flexural, and tensile strengths. The details of the tests performed on the concrete at fresh and hardened states are explained in the next sections.

### 2.3. Mixing and Testing

#### 2.3.1. Mixing

Mixing was done following the requirements of BS 1881-125: 1986. After mixing the dry aggregates and CR for 2 min, the cementitious materials and half of the mixing water were added. After mixing for two more minutes, the remaining water containing the superplasticizer was added and mixed for an additional two minutes to ensure a consistent and homogenous mix.

#### 2.3.2. Workability Tests

The workability of the mixes was assessed using the slump flow, L-box, and V-funnel tests following the specifications of EFNARC 2005.

- Slump flow test: This was performed by filling a slump cone placed at the center of two concentric circles marked on a nonabsorbent platform. The slump cone was then removed, and the time taken for the mixture to reach a 500 mm spread circle was recorded as T_500_ flow time. The maximum diameter of flow spread was also recorded in two directions at 90° as slump flow (d_max_).
- L-box test: This is done to test the concrete’s ability to pass through obstacles without the constituents separating. The passing ability is calculated by dividing the concrete height at the end of the horizontal segment by the height of the remaining concrete in the vertical section. The blocking ratio must not be less than 0.8, and it is calculated using the formula [44]:
  $$\frac{\mathrm{Height}\;\mathrm{of}\;\mathrm{the}\;\mathrm{concrete}\;\mathrm{at}\;\mathrm{the}\;\mathrm{end}\;\mathrm{of}\;\mathrm{horizontal}\;\sec\mathrm{tion}\;\left(H2\right)}{\mathrm{Remaining}\;\mathrm{height}\;\mathrm{in}\;\mathrm{the}\;\mathrm{vertical}\;\sec\mathrm{tion}\;\left(H1\right)}\;\geq \;0.8.$$
- V-funnel test: This is used to test the viscosity and filling ability of the SCC mixture. The trap door at the bottom of the funnel is closed, and the fresh mix is filled into the funnel without tamping. Once the concrete mixture is level at the top of the funnel, the trap door is opened, and the time taken for the mixture to discharge is recorded as the flow time.

#### 2.3.3. Mechanical Properties Tests

- Compressive strength: For each of the 20 mixes developed, three 100-mm cube samples were cast and tested at 7, 14, and 28 days of curing based on the requirements of BS 1881: Part 116:1983. The cubes were subjected to a gradually increasing axial load using the 300-kN universal testing machine until failure. The average of the three results was reported as the compressive strength of the mix at that particular curing day.
- Direct tensile strength test: The tensile strength of the concrete mixes was determined using a dog-bone specimen having a dimension of 420 × 120 × 30 mm^3^, based on the provisions of the Japan Society of Civil Engineers (JSCE). Using a universal testing machine (UTM), a direct tensile load was applied through the axis of the sample at a rate of 0.15 mm/ minute until failure. The average of three results was reported as the tensile strength of the mix at 28 days accordingly.
- Flexural strength: Using three 500 × 100 × 25 mm^3^ beam samples at 28 days of curing, the flexural strength of the mixes was determined using the center point testing method based on the ASTM C78/C78M requirements at a loading rate of 5 mm/ min. The average of three results was reported as the flexural strength of the mix at 28 days accordingly.

## 3. Results

### 3.1. Workability Properties

**Figure 3:**
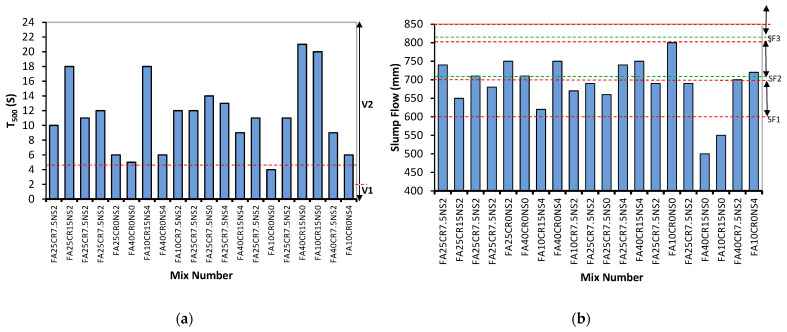
(**a**) T_500_ and (**b**) slump flow (d_max_).

**Figure 4:**
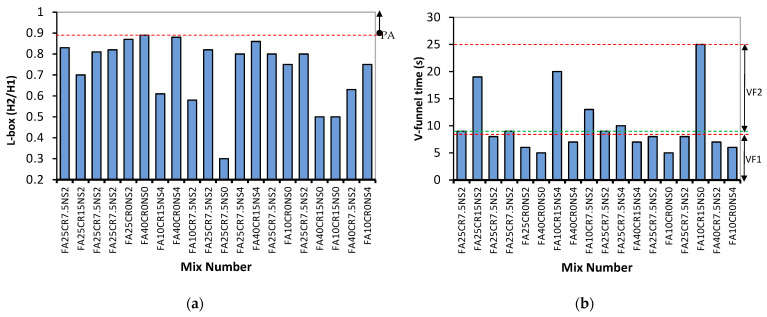
(**a**) L-box (passing ability) and (**b**) V-funnel.

Figure 3 and Figure 4 present the results of the workability tests conducted on the RSCC. Figure 3a shows the T_500_ for all the mixes ranging between 4 and 21 s. Hence, for having a T_500_ of more than 2 s, all the mixes fall within the V2 viscosity class based on the EFNARC classification [54]. The T_500_ results show that the FA significantly reduced the viscosity of the mix, making it spread to 500 mm at a lower time. FA10CR0NS0 has the lowest T_500_ of all the mixes, at 4 s, and comprises FA solely without CR or SF. It is followed by FA40CR0NS0, having a T_500_ of 5 s. It is reported that FA increases the workability of cement composites through the “ball bearing” effect due to the nature of its smooth spherical particles [55]. However, with the addition of NS, an increase in the T_500_ was observed. FA25CR0NS2 and FA10CR0NS04 have a 33.33 and 16.67% increase in the T_500_ compared to FA10CR0NS0 and FA40CR0NS0, respectively. The inclusion of NS increases the viscosity of the mixtures, resulting in a higher T_500_ due to the tiny size and large surface area of its particles, which increases the water demand, as described by Guneyisi et al. [52]. The T_500_ drastically increased with an increase in the CR replacement, indicating a reduction in the viscosity of the concrete. Mixes having the highest CR of 15% exhibited the highest T_500_, as can be observed from FA10CR15NS0, FA40CR15NS0, FA10CR15NS0, FA25CR15NS2, and FA10CR15NS4. It is attributed to the CR particles’ rough surface texture, leading to internal friction that significantly reduces the fresh concrete flow rate, as reported by Wanasinghe et al. [3].

The slump flow is presented in Figure 3b. The SF ranges between 500 to 800 mm, with mixes falling within the three SF classifications (SF1, SF2, and SF3) by EFNARC 2005. The mix with the highest SF is SF10CR0NS0, having only FA among the three variables. FA40CR0NS4 and FA25CR0NS2 were two more mixes with significant FA levels with high SF values. This agrees with the earlier discussion on the effect of FA on the workability of cement composites. The addition of NS and CR led to a decrease in the flowability of most of the mixes. As can be observed, all the mixes having a 15% CR fall within the low flowability class (SF1). The remaining mixes fall within the SF2 class, with only mix number F10CR0NS0 falling within the SF3 category. The negative influence of the CR on the viscosity and flowability of the SCC is due to the lower density of the rubber particles than the fine aggregate particles they replace, which impacted the capacity of the mix to flow under its weight, delaying its ability to spread fast, as reported by Ismail et al. [56].

**Figure 5:**
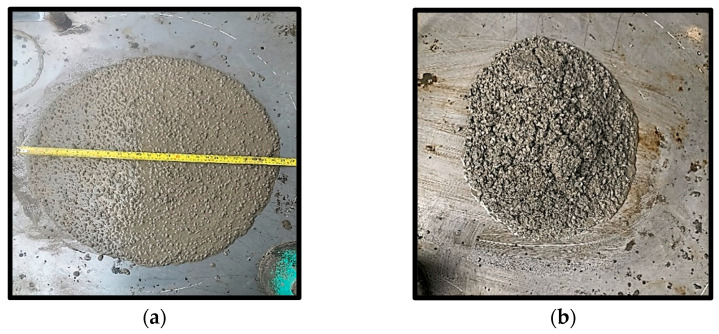
Slump flow for (**a**) mix FA25CR7.5NS0 and (**b**) mix FA10CR15NS0.

The L-box result shown in Figure 4a presents the mixes’ passing ability (PA). EFNARC 2005 specifies two classes (PA1 and PA2) based on the ease of flow of the SCC as indicated by the red line in Figure 4a. Similar to previous results, all mixes with high CR replacement (7.5–15%) have values below 0.8 corresponding to the PA2 class. Furthermore, the V-funnel test results in Figure 4b follow the same pattern as the other tests discussed. Mix number FA10CR15NS0 with the lowest FA content and highest CR has the highest viscosity with a VF of 25 s. In addition, mix numbers FA40CR0NS0 and FA10CR0NS0 have the lowest viscosity with a flow time of 5 s. The increase in the VF time with CR is ascribed to the lower density of the rubber particles, reducing the mix’s ability to flow out freely due to gravity. Similarly, the NS generally reduced the workability by its filling action and by absorbing so much water by its large surface area, increasing the mix’s cohesiveness and viscosity [49]. The positive effect of high FA replacement and lower CR on the workability of the mixes can be observed in how flowable and consistent Mix number FA25CR75NS0 turned out to be as shown in Figure 5a. However, at a reduced FA and higher CR replacements, the mix turned out to have poor flowability as shown in Figure 5b.

### 3.2. Mechanical Properties

#### 3.2.1. Compressive Strength

**Figure 6:**
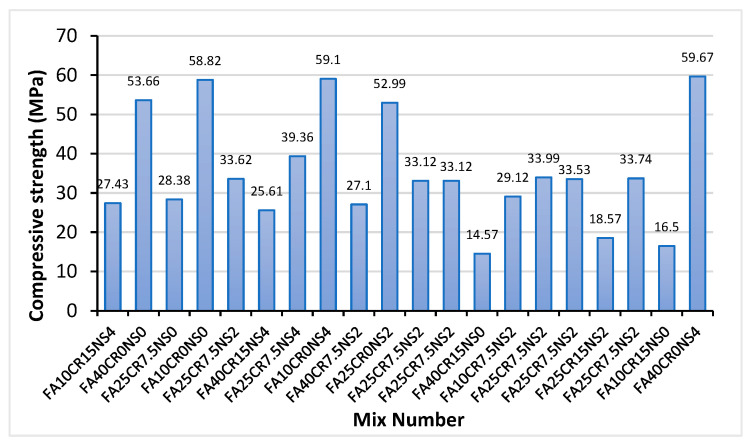
Compressive strength of RSCC mixes at 28 days.

Figure 6 shows the compressive strength of all the SCC mixes at 28 days. The compressive strength ranges from 14.57 MPa for FA40CR15NS0 to 59.67 MPa for FA40CR0NS4. The compressive strength decreases with an increase in CR. This can be observed from all mixes with 7.5 to 15% CR, having relatively lower compressive strength. However, with the exceptions of FA10CR15NS0 and FA40CR15NS0, all the mixes attained the minimum compressive strength for structural concrete specified by ACI 318. This is due to the inadequate bonding between the CR and cement matrix and the CR particles’ low stiffness [48]. As the load is applied, there is no compatibility of strains between the CR and the hardened cement paste at the interfacial transition zone (ITZ), leading to stress concentration and crack forming around the CR particles, being the weak points as confirmed by many researchers [8,43,57,58]. However, with adequate amounts of other variables (FA and NS), an RSCC with a strength above 30 MPa can be produced with 7.5% CR replacements.

On the other hand, the influence of FA on compressive strength can be seen in mixes with only FA (no CR and NS), such as FA10CR0NS0 and FA40CR0NS0, which have compressive strengths of 58.82 Mpa and 53.66 Mpa, respectively. FA particles being smaller than cement have a filling effect resulting in the densification and increased compact microstructure of the concrete, increasing the strength. Furthermore, the pozzolanic effect of FA increases the compressive strength, especially at lower dosage due to the secondary hydration reaction, as confirmed by Posi et al. [59]. The drop in strength from 58.82 Mpa to 53.66 Mpa when the FA percentage rises from 10% to 40% is attributable to decreased reactivity owing to lower cement content, as explained by Turk and Nehdi [59] on a similar finding.

The addition of NS in the mix significantly increased the compressive strength, especially for mixes with no or low CR amounts. Mixes having the highest NS content of 4% (FA40CR0NS4 and FA10CR0NS4) exhibited the highest compressive strengths of 59.67 and 59.1 MPa, respectively. NS is reported to enhance the compressive strength of cement composites due to its high reactivity with cement hydration products, including Ca(OH)_2,_ to produce calcium-silica-hydrate (C-S-H) gel [43]. Similarly, its small particle size has a filling effect, creating a dense and compact microstructure [60]. In addition, despite the reported effect of FA in slowing the early age strength gain of cementitious composites, findings by many researchers such as Xu and Shi [61] and Mohamed [62] stated that NS adjusts the SiO_2_/AlO_3_ ratio of high volume fly ash concretes, leading to early strength gain. This may be the reason behind FA40CR0NS4 having the highest strength at 28 days despite having a 40% FA replacement. In the same vein, despite the CR replacement levels, all mixes having 2–4% NS content have strengths of more than 17.23 MPa minimum compressive strength for structural concrete specified by ACI 318. This is attributable to the ITZ densification effect of the NS. Mohammed et al. [41] reported that NS densifies the ITZ between CR and cement paste due to its physicochemical effects of pozzolanic reaction and pore filling, leading to better stress transfer and enhancement in compressive strength. As a result of the preceding, it can be established that a structural RSCC may be prepared using up to 25–40% FA replacement of cement at 4% NS addition and 7.5–15% CR replacement of a fine aggregate.

**Figure 7:**
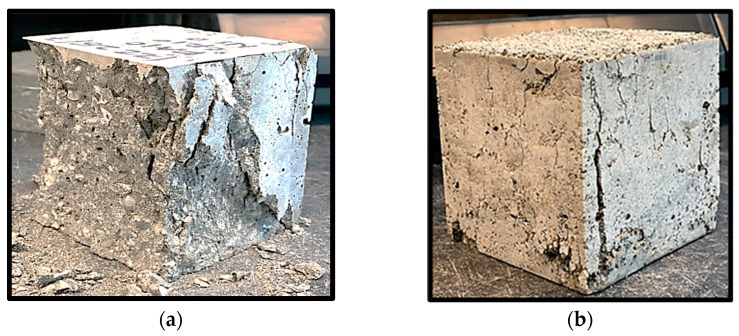
(**a**) FA40CR0NS4 sample at failure; (**b**) FA40CR15NS4 sample at failure.

The failure mechanisms of samples from FA40CR0NS4 and FA40CR15NS4 mixes are shown in Figure 7a,b, respectively. Although FA40CR0NS4 could withstand more significant stress before failing, it broke into pieces in a typical brittle failure pattern when the failure load was reached. However, because of the presence of CR, the FA40CR15NS4 sample failed in a more ductile way at the ultimate stress, exhibiting few cracks. This attests to the effectiveness of the CR in reducing the brittleness of the concrete.

#### 3.2.2. Flexural Strength

**Figure 8:**
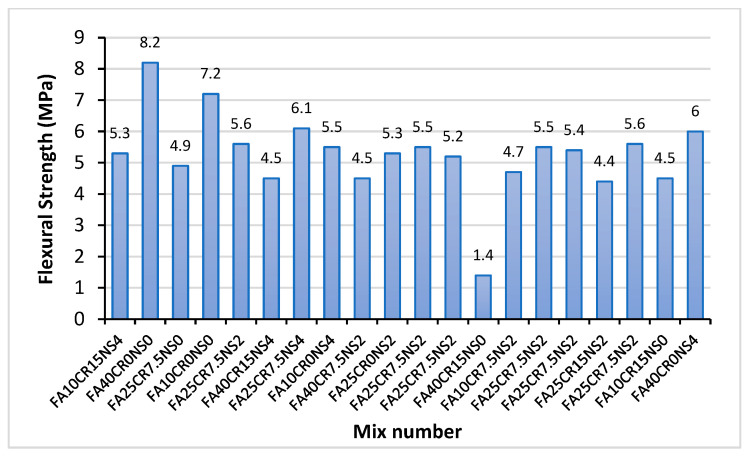
Flexural strength result at 28 days.

The flexural strength values of the RSCC mixes are shown in Figure 8. With the exception of mix FA40CR15NS0 having the lowest strength of 1.4 MPa, all the other mixes have relatively high flexural strength values ranging from 4.4 to 8.2 MPa. These values correlate well with the flexural strength values of SCC containing recycled rubber and silica fume by Busic et al. [48]. However, the trend of the results indicates that the concrete’s flexural strength decreases with an increase in the CR replacement. This is attributed to the lower stiffness of the CR particles compared to the fine aggregate they replaced. After a similar observation, Sharul et al. [43] noted that the CR has lower strength, lower specific gravity, and a different load-bearing capacity than sand particles, resulting in lesser flexural strength. Interestingly, the mix with the highest flexural strength (FA40CR0NS0) and the lowest (FA40CR15NS0) have something in common: They both have 40% FA replacement levels. FA40CR0NS0 was able to attain high strength (8.2 MPa) due to the dual effects of pozzolanic reaction and filler effects of the FA. However, due to the presence of 15% CR in FA40CR15NS0, the interaction of the FA was interrupted by the CR particles owing to their influence on the mixing water, disrupting the hydration process that may give essential products for the pozzolanic reaction to take place. A similar effect was explained by Shahrul et al. [43].

The addition of NS has played a vital role in enhancing the flexural strength of the concrete. An increase of 221% was recorded between FA40CR15NS0 (1.4 MPa) and FA40CR15NS4 (4.5 MPa) as the NS content increased from 0 to 4%. Similarly, an increase of 17.8% between FA10CR15N0 (4.5 MPa) and FA10CR15NS4 (5.3 MPa) was observed. These enhancements in strength with the addition of NS are consistent with prior research findings. As supported by Mohamed [62], Yu et al. [63], and Razavi et al. [60], NS increases the compactness of the composite due to its large surface-to-volume ratio, thereby increasing the concrete’s flexural toughness, leading to higher strength. Similarly, Gao et al. [64] reported that the use of NS increases the flexural fatigue performance of concrete due to its secondary reaction and filling the micropores. In the same vein, the enhanced reactivity of the NS increases the production of C-S-H gel, which fills the ITZ between the aggregates and the hardened cement paste increasing the stress transfer under load. This explains why mixes with CR and NS perform better than their counterparts without NS.

**Figure 9:**
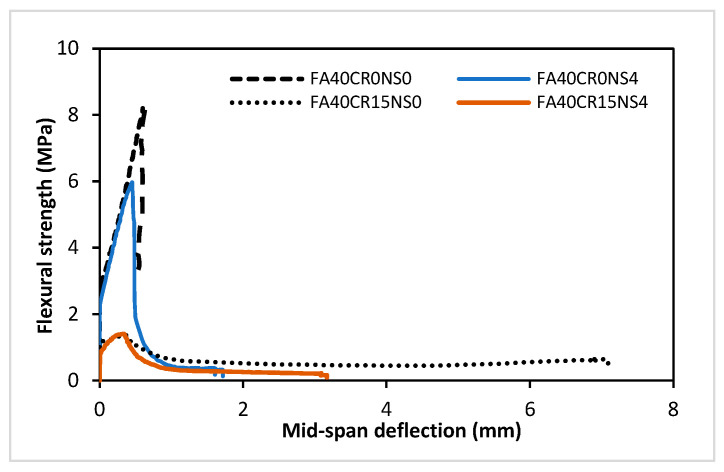
Stress-deflection curve for flexural test.

As presented in the stress deflection curves in Figure 9, the mid-span deflection increases with an increase in CR replacement. This is attributed to the effect of CR in reducing the brittleness of the composites due to its lower elastic modulus as documented in previous studies [13]. On the other hand, the presence of FA and NS in the mix makes it more brittle as can be observed from the curves. This is attributed to the reactivity of the two pozzolans, creating a more dense, compact, and strong but brittle microstructure.

#### 3.2.3. Tensile Strength

**Figure 10:**
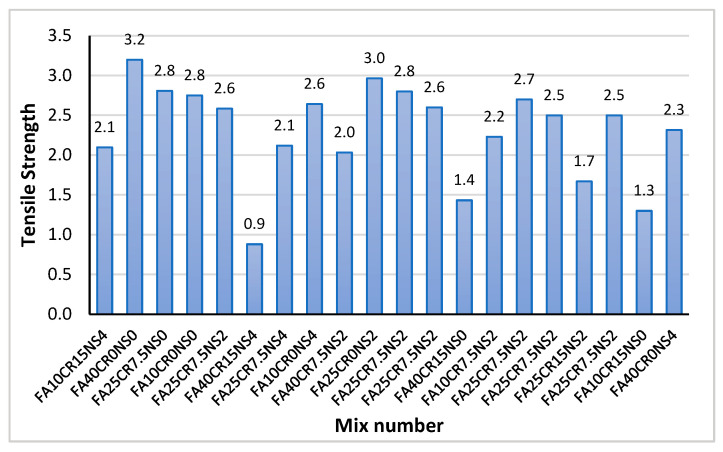
Tensile strength of RSCC mixes at 28 days.

Figure 10 shows the results of the direct tensile test. The values range from 0.9 to 3.2 MPa and are comparable with the findings from previous research [9]. A general decline in the tensile strength with CR is observed across almost all the mixes. Mix FA40CR15NS4 at 15CR has the lowest tensile strength of all mixes (0.9 MPa). This is in sharp contrast to a similar mix without CR (FA40CR0NS4) having a 155.6% higher tensile strength. Similarly, between FA0CR15NS4 and FA0CR0NS4, FA25CR0NS2 and FA25CR15NS2, and FA10CR15NS0 and FA10CR0NS0, there was a 23.8 percent, 76 percent, and 115 percent increase, respectively. The CR, as explained earlier, affects the strength of the composite due to its lower stiffness and the poor bonding at the ITZ, leading to stress concentration and crack propagation around the particles [65].

With the addition of NS in the mix, there are noticeable improvements in the strength. Almohammad-albakkar and Behfarnia [49] reported that NS increases the bonding between the aggregates and cement matrix. A similar conclusion was reached by Shahrul et al. [43]. However, despite the high amount of NS, the low strength of FA40CR15NS4 is worth noting. It has been reported from previous studies that a high quantity of NS can sometimes affect the strength of concrete due to the tendency of the particles to agglomerate and make dispersion difficult [63]. Similarly, as explained by Nandhini and Ponmalar [47], the use of a high amount of fine reactive materials can lead to the production of large crystals of Ca(OH)_2_ that weakens the ITZ and affects the strength. In addition, it is worth mentioning that the high amount of FA in the mix is likely to lower the strength due to the reduction in the cement content, as explained by Abdulkadir et al. [18].

## 4. FESEM Analysis

**Figure 11:**
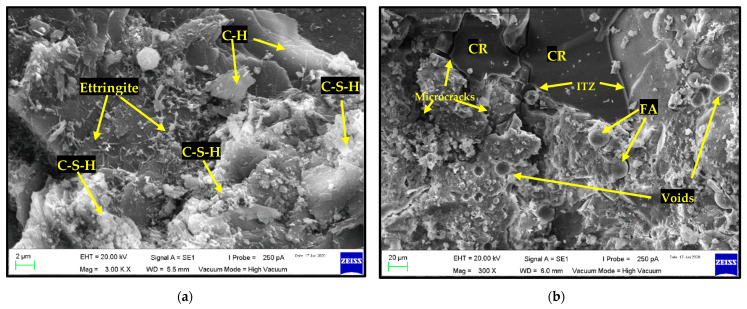
(**a**) Formation of primary and secondary hydration products due to FA and NS. (**b**) Presence of voids and microcracks in a mix having CR.

The FESEM images of some selected mixes are presented in Figure 11a,b. The FESEM reveals magnified views of the morphology of the mixtures, allowing for easy identification of the composition and distinct physical and chemical phases of the ingredients. Such features include the cement-hydration products and the interfacial transition zones (ITZs) between the hardened cement paste (HCP) and the aggregates (fine, coarse, and CR). As can be observed from Figure 11a, the presence of FA and NS has led to the production of more primary and secondary hydration products, leading to the enhanced strength of the composites without CR. As a result, all mixes having only FA and NS, such as FA25CR0NS2 and FA10CR0NS2, gained strength faster. However, Figure 11b shows a mix containing CR. It can be observed that the interface (ITZ) between the CR and hardened paste is relatively smaller than that presented by Najim and Hall for untreated CR [34]. This is attributed to the enhanced reactivity, pore-refining, and densifying effects of the NS, as reported by Shahrul et al. [43] and Mohammed et al. [41]. Nonetheless, despite improvements in the ITZ, concrete failure frequently occurs near the CR due to the lower density and stress concentration leading to microcrack formation, as shown in Figure 11b.

## 5. RSM Modeling and Optimization

### 5.1. Response Surface Models Development and ANOVA

The goal of employing the RSM is to develop response surface models and analyze the developed models using analysis of variance (ANOVA). The mechanical strengths of the developed rubberized self-compacting concrete (RSCC) mixes were considered for RSM modeling and optimization. In this case, the quadratic models were deemed more suitable for all three responses (compressive, flexural, and tensile strengths) and are presented in coded terms in Equations (1)–(3). The Equations in terms of coded factors may be used to predict response for different amounts of each variable. The high levels of the factors are represented as +1 by default, whereas the low levels are −1. By comparing the factor coefficients, the coded Equation may be used to determine the relative importance of the variables. A, B, and C represent the input factors (FA, CR, and NS). Table 4 summarizes the ANOVA results performed.

The ANOVA was performed at a 95% level of significance, and any model or model term having a probability of less than 5% is considered significant. In this regard, all the developed models are significant for having a probability of less than 0.05. Considering the individual model terms, C, BC, B^2^, and C^2^ are statistically significant for the compressive strength model. Similarly, B, AB, and BC are the significant model terms for the flexural strength model. The significant model terms for the tensile strength model are B, AB, AC, and A^2^.

One of the essential measures of the quality of a model is the coefficient of determination (R^2^). The R^2^ assesses the model’s fit to the empirical data, ranging from 0 to 1 or a percentage (0 to 100%). Higher values indicate better fit and vice versa. The R^2^ and the other model validation parameters are presented in Table 5. It can be seen that all the models have a high R^2^ of 99%, 87%, and 96% for compressive, flexural, and tensile strength models, respectively. Furthermore, the signal-to-noise ratio is measured by “Adeq. Precision.” A ratio larger than four is preferred. As can be observed from Table 5, the Adeq. Precision values are 30.502, 12.722, and 20.341 for the compressive, flexural, and tensile strength models, respectively. These values indicate that the models are good and can be used for response prediction with reasonable accuracy.

(1) $$CS=+32.25-1.04\times A-18.16\times B+3.92C+0.11\times AB+0.73\times AC+1.96\times BC-2.25\times A^{2}+5.42\times B^{2}+3.51\times C^{2}$$

(2) $$FS=+5.28-0.26\times A-1.20\times B+0.11\times C-0.67\times AB+0.22\times AC+0.98\times BC-0.37\times A^{2}-0.13\times B^{2}+0.49\times C^{2}$$

(3) $$TS=+2.56-0.11\times A-0.65\times B-0.14\times C-0.15\times AB-0.26\times AC+0.16\times BC-0.34\times A^{2}-0.15\times B^{2}-6.30\times 10^{-3}\times C^{2}$$

where CS, FS, and TS are the compressive, flexural, and tensile strength, respectively.

**Figure 12:**
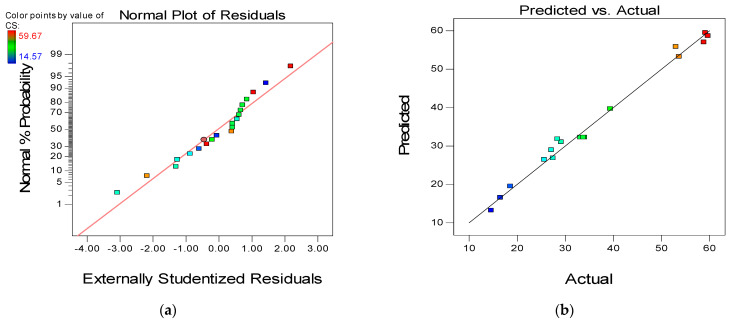
(**a**) Normal plot of residuals and (**b**) predicted versus actual plot for compressive strength.

**Figure 13:**
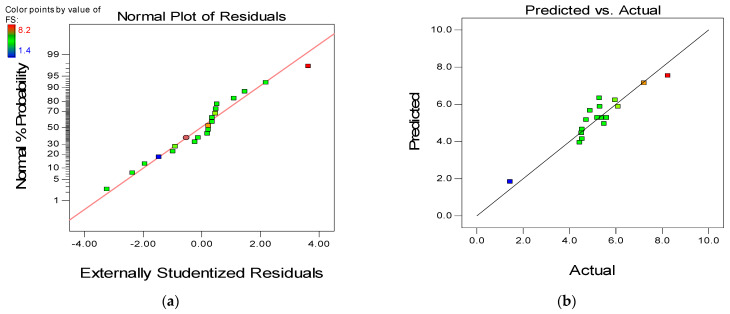
(**a**) Normal plot of residuals and (**b**) predicted versus actual plot for flexural strength.

**Figure 14:**
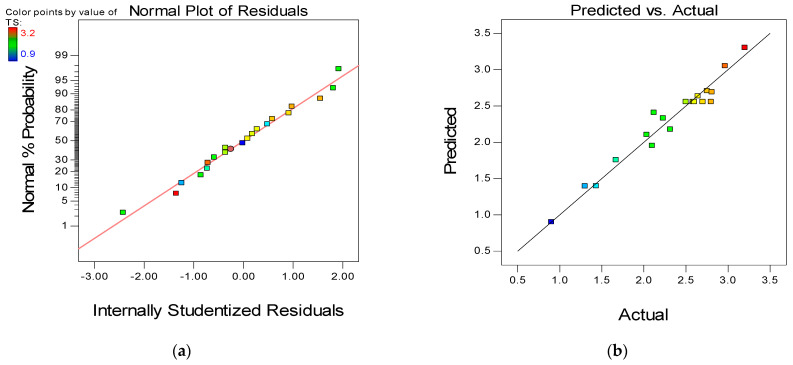
(**a**) Normal plot of residuals and (**b**) predicted versus actual plot for tensile strength.

To further assess the quality and adequacy of the developed response models, two of the powerful model diagnostic tools employed are the “Normal versus Residual” plot and the “Actual versus Predicted plot” as presented in Figure 12, Figure 13 and Figure 14, respectively for the three models (CS, FS, and TS). The linearity of the data points along the fit line illustrates the developed models’ quality in all graphs. The pattern of the data points on the normal plots of residuals indicates that the error terms are normally distributed, which is desirable. The residuals are normally distributed if 95% of the points lie between −2 and +2, as is the case for all the developed models [66].

**Figure 15:**
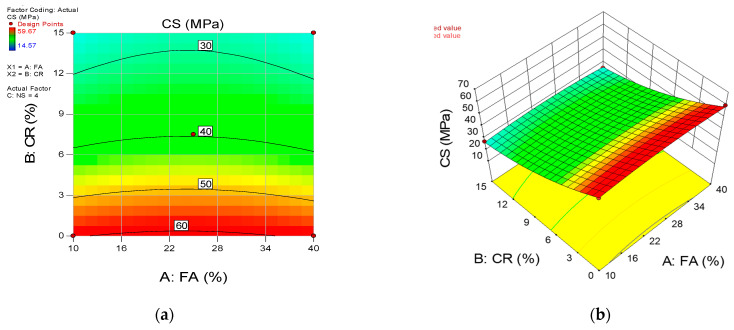
(**a**) 2D contour plot and (**b**) 3D response surface diagram for CS.

**Figure 16:**
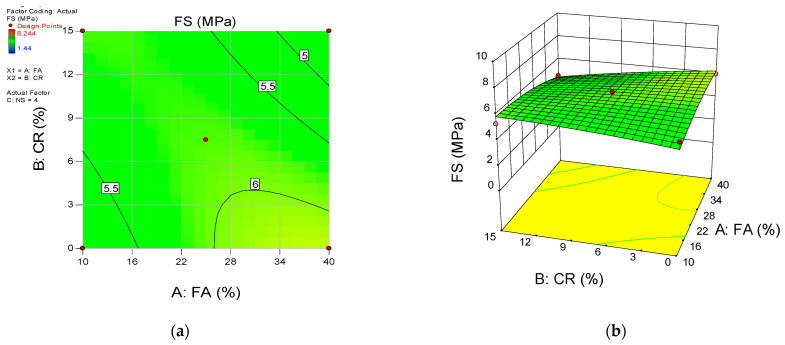
(**a**) 2D contour plot and (**b**) 3D response surface diagram for FS.

**Figure 17:**
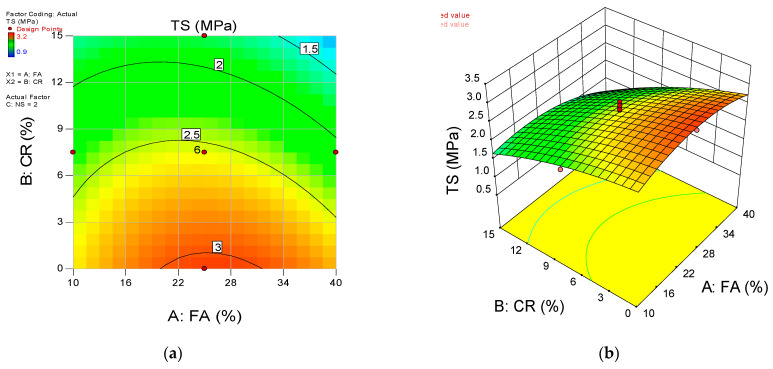
(**a**) 2D contour plot and (**b**) 3D response surface diagram for TS.

The influence of the input variables’ interaction on the responses is presented using the 2D contour plots and the 3D response surface diagrams. Figure 15, Figure 16 and Figure 17 show the response surface diagrams for the CS, FS, and TS models, respectively. For a three-input variables situation such as the current research, the interaction of two variables is represented while keeping the third variables constant. In this scenario, the 2D contour plots and 3D response diagrams for the interaction between the FA and CR are provided at a constant value of the NS (set at the highest level of 4 percent).

The diagrams’ color coding represents the response’s magnitude and the varying levels of the input variables under consideration. Figure 15a,b shows the 2D contour and 3D response diagrams for compressive strength, respectively. The plots indicate a high intensity of compressive strength across all levels of FA while there is a significant decrease in the strength values with an increase in the CR at a constant NS of 4%. As explained earlier, this is due to the void-filling and densification effects and the pozzolanic reaction of the FA and NS. On the other hand, the loss in the mechanical strengths is attributed to the lower density of the CR and lack of proper bonding with the hardened cement matrix. Similar behavior can be observed from the remaining response surface diagrams of the flexural and tensile strengths.

### 5.2. Optimization

An optimization attempt is conducted to find appropriate levels of the independent variables to achieve an optimum level of the response of interest. This is accomplished by establishing goals for the variables (input factors and response) with varying criteria and levels of importance in order to achieve the objective function. The desirability value (0 ≤ *d_j_* ≤ 1) is used to evaluate the optimization. The greater the value’s proximity to one, the better the outcome [67].

**Figure 18:**
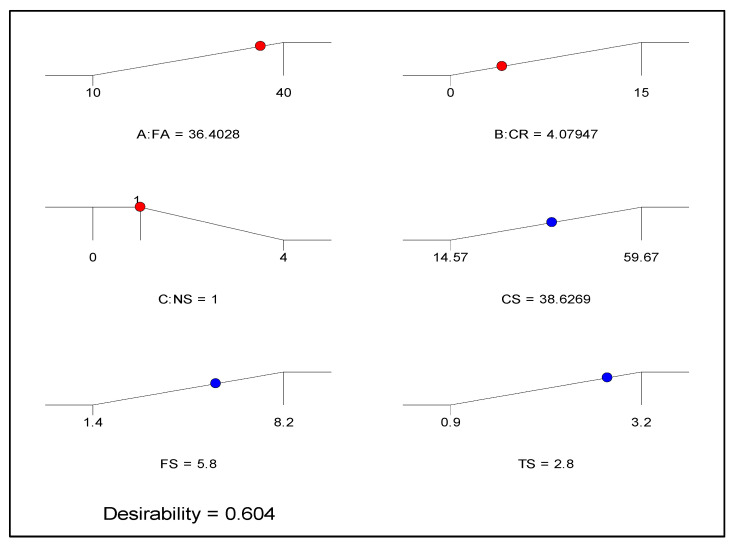
Optimization solution ramp.

**Figure 19:**
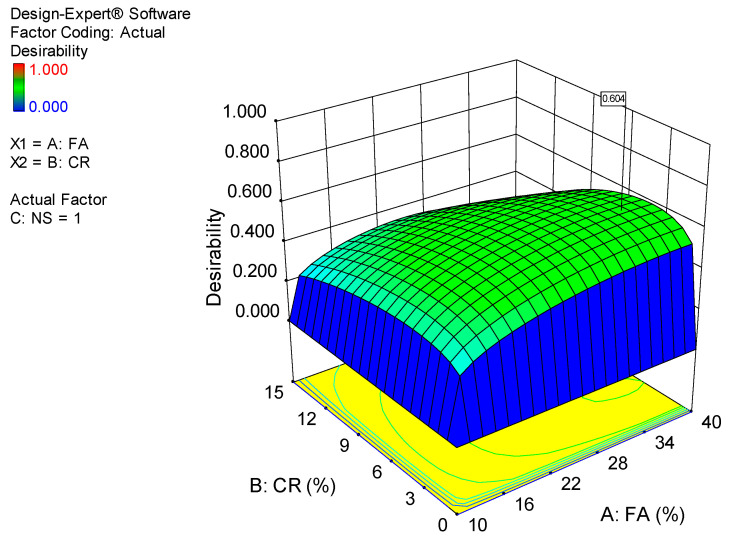
3D response surface diagram for the desirability of the optimization.

The optimization goals and criteria set in this case are presented in Table 6. The objective of the optimization is to maximize all three responses, as shown in the table. Similarly, CR and FA are waste materials, and their use is being encouraged in cementitious composites; hence, the objective is to maximize their use. On the other hand, the use of NS has been minimized within a range of 1 to 4% so that the system can select the best level to achieve the overall goal. After running the optimization, the results revealed that the maximum values of 38.63 MPa, 5.8 MPa, and 2.8 MPa could be obtained for the CS, FS, and TS, respectively, at an optimum level of 36.38%, 4.08%, and 1% for FA, CR, and NS, respectively. The optimization’s desirability is determined to be 60%, which is highly reasonable given the nature of the vast variabilities in the response values. The optimization solution and the 3D response surface diagram for the desirability are presented in Figure 18 and Figure 19.

Finally, experimental validation was performed by producing RSCC samples using the optimum input variables recommended by the optimization to determine the CS, FS, and TS values at 28 days. Table 7 presents the results of the test performed. This shows that the developed response models are strong and can be used to predict the responses with an acceptable level of accuracy. The experimental error between the experimental and predicted results was calculated for all the responses using Equation (4), and the results are found to be less than 5%, which is the acceptable limit, as presented in Table 7.

(4) $$\delta =\left|\frac{\vartheta_{E}-\vartheta_{P}}{\vartheta_{P}}\right|\times 100\%\;$$

where δ, $\vartheta_{E}$, and $\vartheta_{P}$ represent the percentage error, as well as the experimental and predicted values, respectively.

## 6. Conclusions

This research was performed to determine the effect of CR and a ternary blend of cement, FA, and NS on the fresh and mechanical properties of SCC. The following conclusions were drawn at the end of the investigations:

1. In the fresh state, the RSCC workability properties measured through T_500_, slump flow, L-box, and V-funnel tests increased with an increase in the FA replacement due to its smooth spherical particles, while the opposite effect was observed with CR replacement and NS addition. Due to the rough surface texture and low density of the rubber particles, CR caused the most significant loss in all the workability parameters. The reduction in the workability with the NS addition is ascribed to the large surface area of the particles, which absorbed much of the mixing water, reducing the mix’s fluidity.
2. Increases in NS and modest levels of FA boosted the concrete’s compressive, flexural, and tensile strengths, which was ascribed to the physicochemical effects of the NS and FA, resulting in a dense and compact microstructure. On the other hand, increases in the CR resulted in a considerable reduction in the concrete’s mechanical strengths, which was attributed to the CR’s low stiffness and poor bonding at the CR-cement interface. However, at all CR replacement levels, all mixes containing NS attained the compressive strength beyond the minimum specified by ACI318 for structural concrete due to its enhancement of the bonding between the CR and hardened cement paste at the ITZ. In addition, the brittleness of the concrete is reduced owing to the CR.
3. A closer look at the interaction of the FA, NS, and CR through FESEM revealed a formation of more S-C-H gel and densification of the concrete microstructure due to the FA and NS. In addition, the densification of the ITZ between the CR and cement paste by the NS was observed. Similarly, the images revealed the propagation of microcracks around the CR due to the stress concentration.
4. Response surface models for predicting the mechanical strengths were developed and validated using ANOVA. The models have R^2^ values of 99%, 87%, and 96% for CS, FS, and TS, respectively. An optimization produced 36.38%, 4.08%, and 1.0% for the optimum FA, CR, and NS replacement levels at a desirability value of 60%. Experimental validation demonstrated a strong correlation between predicted and experimental results, with a percentage error of less than 5% for all three responses analyzed.
5. It is evident from the results that a rubberized self-compacting concrete with FA and NS can be produced to have good workability properties in the fresh state and appreciable mechanical strengths at the hardened state.

## Figures and Tables

**Table 1:** Physical properties of aggregates and CR.

Physical Properties	Fine Aggregates	Coarse Aggregates	Crumb Rubber
Specific Gravity	2.65	2.61	0.95
Water Absorption (%)	2.10	1.17	-
Moisture Content (%)	1.3	-	-
Fineness Modulus	2.20	6.127	0.92

**Table 2:** Oxide composition of the OPC and FA used.

Oxide	CaO	SiO_2_	Fe_2_O_3_	Al_2_O_3_	K_2_O	MgO	SO_3_	Na_2_O
OPC	64.64	21.28	3.36	5.60	1.68	2.06	2.14	0.98
FA	5.98	64.69	4.90	18.89	1.14	1.99	0.10	2.41

**Table 3:** RSM generated mixes and proportions of materials.

Mix Reference	Cementitious Material					Aggregates				Water kg/m^3^
	OPC kg/m^3^	FA		NS		Fine Agg.	CR		Coarse Agg.	
		%	kg/m^3^	%	kg	kg/m^3^	%	kg/m^3^	kg/m^3^	
FA10CR0NS4	540.00	10	60.00	4	0.02	882.00	0	0.00	588.00	210.00
FA10CR15NS0	540.00	10	60.00	0	0.00	749.70	15	132.30	588.00	210.00
FA10CR7.5NS2	540.00	10	60.00	2	0.01	815.85	7.5	66.15	588.00	210.00
FA10CR0NS0	540.00	10	60.00	0	0.00	882.00	0	0.00	588.00	210.00
FA10CR15NS4	540.00	10	60.00	4	0.02	749.70	15	132.30	588.00	210.00
FA25CR7.5NS2	450.00	25	150.00	2	0.01	815.85	7.5	66.15	588.00	210.00
FA25CR0NS2	450.00	25	150.00	2	0.01	882.00	0	0.00	588.00	210.00
FA25CR7.5NS2	450.00	25	150.00	2	0.13	815.85	7.5	66.15	588.00	210.00
FA25CR7.5NS4	450.00	25	150.00	4	0.02	815.85	7.5	66.15	588.00	210.00
FA25CR15NS2	450.00	25	150.00	2	0.01	749.70	15	132.30	588.00	210.00
FA25CR7.5NS2	450.00	25	150.00	2	0.01	815.85	7.5	66.15	588.00	210.00
FA25CR7.5NS0	450.00	25	150.00	0	0.00	815.85	7.5	66.15	588.00	210.00
FA25CR7.5NS2	450.00	25	150.00	2	0.01	815.85	7.5	66.15	588.00	210.00
FA25CR7.5NS2	450.00	25	150.00	2	0.01	815.85	7.5	66.15	588.00	210.00
FA25CR7.5NS2	450.00	25	150.00	2	0.01	815.85	7.5	66.15	588.00	210.00
FA40CR0NS4	360.00	40	240.00	4	0.02	882.00	0	0.00	588.00	210.00
FA40CR0NS0	360.00	40	240.00	0	0.00	882.00	0	0.00	588.00	210.00
FA40CR7.5NS2	360.00	40	240.00	2	0.01	815.85	7.5	66.15	588.00	210.00
FA40CR15NS0	360.00	40	240.00	0	0.00	749.70	15	132.30	588.00	210.00
FA40CR15NS4	360.00	40	240.00	4	0.02	749.70	15	132.30	588.00	210.00

**Table 4:** ANOVA.

Response	Source	Sum of Squares	Df	Mean Square	F-Value	*p*-Value > F	Significance
Compressive strength (MPa)	Model	3724.12	9	413.79	90.06	<0.0001	Yes
	A-FA	10.73	1	10.73	2.34	0.1574	No
	B-CR	3296.4	1	3296.4	717.46	<0.0001	Yes
	C-NS	153.98	1	153.98	33.51	0.0002	Yes
	AB	0.088	1	0.088	0.019	0.8926	No
	AC	4.26	1	4.26	0.93	0.3581	No
	BC	30.73	1	30.73	6.69	0.0271	Yes
	A^2^	13.88	1	13.88	3.02	0.1129	No
	B^2^	80.89	1	80.89	17.61	0.0018	Yes
	C^2^	33.95	1	33.95	7.39	0.0216	Yes
	Residual	4.02	10	0.4			
	Lack of Fit	45.35	5	9.07	75.65	0.0001	Yes
	Pure Error	0.6	5	0.12			
	Cor Total	3770.06	19				
Flexural strength (MPa)	Model	27.58	9	3.06	7.63	0.0019	Yes
	A-FA	0.66	1	0.66	1.64	0.2287	No
	B-CR	14.29	1	14.29	35.55	0.0001	Yes
	C-NS	0.11	1	0.11	0.28	0.6066	No
	AB	3.64	1	3.64	9.07	0.0131	Yes
	AC	0.39	1	0.39	0.96	0.3503	No
	BC	7.7	1	7.7	19.16	0.0014	Yes
	A^2^	0.37	1	0.37	0.92	0.3603	No
	B^2^	0.048	1	0.048	0.12	0.7361	No
	C^2^	0.67	1	0.67	1.67	0.2254	No
	Residual	4.02	10	0.4			
	Lack of Fit	3.91	5	0.78	36.09	0.0006	Yes
	Pure Error	0.11	5	0.022			
	Cor Total	31.6	19				
Tensile strength (MPa)	Model	6.47	9	0.72	25.79	<0.0001	Yes
	A-FA	0.13	1	0.13	4.66	0.0562	No
	B-CR	4.19	1	4.19	150.24	<0.0001	Yes
	C-NS	0.2	1	0.2	7.21	0.0229	Yes
	AB	0.18	1	0.18	6.31	0.0308	Yes
	AC	0.56	1	0.56	19.92	0.0012	Yes
	BC	0.2	1	0.2	7.12	0.0236	Yes
	A^2^	0.32	1	0.32	11.31	0.0072	Yes
	B^2^	0.065	1	0.065	2.32	0.1588	No
	C^2^	1.09 × 10^−4^	1	1.09 × 10^−4^	3.92 × 10^−3^	0.9513	No
	Residual	0.28	10	0.028			
	Lack of Fit	0.21	5	0.042	3.04	0.124	No
	Pure Error	0.069	5	0.014			
	Cor Total	6.75	19				

**Table 5:** Parameters for model verification.

Model Validation Parameters	CS	FS	TS
Std. Dev.	2.14	0.63	0.17
Mean	35.60	5.28	2.31
C.V. %	6.02	12.02	7.24
PRESS	295.93	43.98	2.08
−2 Log Likelihood	73.39	24.66	−28.70
R-Squared	0.99	0.87	0.96
Adj R-Squared	0.98	0.76	0.92
Pred R-Squared	0.92	−0.39	0.69
Adeq Precision	30.50	12.72	20.34
BIC	103.35	54.62	1.26
AICc	117.84	69.10	15.74

**Table 6:** Optimization goals and results.

Factors		Input Factors			Responses (Output Factors)		
		FA (%)	CR (%)	NS (%)	CS (MPa)	FS (MPa)	TS (MPa)
Value	Minimum	10	0	0	14.57	1.4	0.9
	Maximum	40	15	4	59.67	8.2	3.2
Goal		Maximize	Maximize	Minimize	Maximize	Maximize	Maximize
Optimization result		36.38	4.08	1.00	38.63	5.80	2.80
Desirability		0.604 (60%)					

**Table 7:** Experimental validation.

Response	Predicted	Experimental	Error (%)
CS (MPa)	38.63	40.10	3.8
FS (MPa)	5.80	5.58	3.9
TS (MPa)	2.80	2.92	4.2

## Data Availability

All data contained within the article.
